# An SDT-informed online intervention supporting children's home-based exercise: A randomized controlled trial

**DOI:** 10.1016/j.jesf.2026.200478

**Published:** 2026-04-27

**Authors:** Chi-Ching Gary Chow, Fenghua Sun, Yu-Hin Kong

**Affiliations:** Department of Health and Physical Education, The Education University of Hong Kong, Hong Kong

## Abstract

This randomized controlled trial evaluated the effectiveness of a Self-Determination Theory (SDT)-informed online intervention in promoting moderate-to-vigorous physical activity (MVPA) among Hong Kong primary school students. Over two academic years (2022 – 24), 119 child–guardian dyads were randomly assigned to: (1) Exercise Only, (2) Coaching Only, (3) Exercise-plus-Coaching, and (4) a Waitlist Control. For eight weeks, guardians accessed private Facebook groups, receiving either exercise videos, SDT-informed coaching cues, or both.

MVPA (ActiGraph) and motivation (SDT scales) were assessed at Baseline (T1), Post-intervention (T2), and Follow-up (T3). Significant group × time interaction effects indicated that both Exercise Only and Exercise-plus-Coaching groups showed increases in MVPA from T1 to T2 (small to medium effect sizes). However, these gains diminished by T3. Similarly, intrinsic motivation and identified regulation were better maintained in the intervention groups with coaching cues, whereas the Waitlist Control showed declines by T2 and T3. Improvements in autonomy and relatedness satisfaction occurred only in the groups involving exercise videos.

Findings demonstrated the short-term efficacy of guardian-led, SDT-informed digital interventions in buffering declines in physical activity and motivation. The value of autonomy-supportive environments and parental involvement is underscored as facilitators of acute engagement. This research contributes to the evidence base supporting the potential for digital dissemination while acknowledging the structured support required for effective delivery. Future research should explore more intensive intervention protocols and investigate how the social connectivity and motivational features of platforms like Facebook can enhance long-term maintenance and broader accessibility across diverse populations.

## Highlights

• SDT-informed online intervention led to short-term gains in children's MVPA and motivation.
• Digital coaching cues and activity choices preserved children's autonomy and relatedness needs.
• Guardian involvement mitigated the decline in physical activity and motivation observed in non-intervention settings.
• While motivation was maintained, the loss of MVPA gains at follow-up suggests a need for long-term support strategies.

## 1 Introduction

Globally, many school-age children (5 – 17 years old) do not meet the World Health Organization guideline of ≥60 min of moderate-to vigorous-intensity physical activity (MVPA) per day.^1^ In multi-city comparisons across Asia, adolescents in Tokyo were the only group exceeding the guideline, while Seoul trailed by a narrow margin, underscoring widespread insufficiency.^2^ In Hong Kong, surveillance data indicates that only 8% of children (ages 6–12) meet the MVPA-60 guideline, signaling a notable public health concern.^3^^,^^4^

Children's MVPA is heavily shaped by school-based opportunities, particularly Physical Education (PE) and structured extracurricular activities, which provide organized time, facilities, and peer contexts that facilitate participation.^5^, ^6^, ^7^, ^8^, ^9^, ^10^ Although other factors, such as active commuting and parental encouragement, can support activity levels, these are often examined less consistently and are more variable across households.^11^^,^^12^ The COVID-19 pandemic highlighted this dependency: when access to school and organized recreation was restricted, children's MVPA dropped markedly due to the loss of structured physical-activity (PA) environments.^13^^,^^14^

In this context, home-based exercise emerged as a practical alternative. However, most programs have traditionally targeted adults or clinical populations (e.g., older adults, chronic disease, obesity), with limited evidence regarding the effectiveness of home-based and online PA programs for children.^15^, ^16^, ^17^ Online and social-media-delivered interventions, such as Facebook-based programs, have shown promise for increasing PA and engagement in adults,^18^^,^^19^ with reports of substantial effects, but generalizability to children is uncertain. While social media offers broad reach and low cost,^20^ many existing programs lack a clear theoretical foundation, which may limit their sustained engagement and impact.^21^ Accordingly, to ensure programs have sustained engagement and impact, there is a strong rationale to ground interventions in behavioral theory and to compare theory-based versus atheoretical approaches.^22^

Self-Determination Theory (SDT) posits that supporting three basic psychological needs: autonomy, competence, and relatedness, fosters self-determined forms of motivation linked to better adherence and well-being.^23^, ^24^, ^25^ Meta-analysis evidence associates autonomous motivation with higher physical activity (PA), whereas controlled regulation and amotivation relate negatively.^25^ SDT-informed interventions have shown promise for engagement, for example, prior work using Facebook programs reported high adherence and improved motivational indices, though objective MVPA outcomes can be variable across measures and groups.^26^ In school settings, PE curricula often emphasize physical competence and knowledge,^27^ yet well-intended practices may unintentionally elicit controlled motivation (e.g., via guilt or external rewards), which can undermine participation beyond class time.^28^^,^^29^ Outside school, lower participation may reflect fewer structured opportunities and developing self-regulatory capacity rather than a lack of interest.

To extend active behavior beyond school contexts, out-of-school influences, especially guardians, are salient through encouragement, modelling, and support (e.g., time, space, routines).^30^, ^31^, ^32^ Relatedly, educators' self-determined motivation has been linked to more autonomy-supportive teaching practices,^33^ and online learning environments that support autonomy, competence, and relatedness tend to enhance engagement and persistence.^34^ Parent- or guardian-led interventions have demonstrated feasibility and favorable engagement using take-home materials and simple equipment,^35^^,^^36^ yet there remains a need to systematically examine how guardians can help cultivate home environments that support all three SDT needs: autonomy, competence, and relatedness, rather than focusing on autonomy in isolation, particularly in densely populated urban settings. Framing the guardian's role in creating a supportive home environment represents a promising, yet underdeveloped, avenue for intervention.^37^

Accordingly, this study evaluated a theory-driven, SDT-informed online intervention designed to promote PA among primary school-aged children through guardian-delivered support. This study adopted a multi-arm randomized design to disentangle the contributions of: (i) Exercise Only Group (exercise videos only), (ii) the Coaching Only Group (SDT-informed coaching cues delivered to guardians), and (iii) the Exercise-plus-Coaching Group (the combination of both), compared with (iv) a Waitlist Control Group. This approach enabled tests of overall effectiveness and putative mechanisms of change. Specifically, the objectives were: i) to investigate the effectiveness of online daily workouts for improving PA level at home in primary school-age children, ii) to examine the underlying mechanisms behind the change of PA associated with self-determination theory and iii) to probe the role(s) of guardians behind the change of PA. It is hypothesized that children in intervention groups will show greater improvements in MVPA, motivation, and psychological need satisfaction than those in the Waitlist Control, with the strongest effects in groups receiving guardian support.

## 2 Methods

### 2.1 Participants

Children and their guardians were recruited from seven local primary schools in Hong Kong representing diverse socioeconomic backgrounds. Eligibility screening was conducted by school teachers and a trained research assistant based on predefined inclusion and exclusion criteria. Recruitment took place during the early academic years of 2022/23 and 2023/24. Prior to enrolment, voluntary informed consent was obtained from all guardians. Participation was entirely voluntary, and both children and guardians were informed of their right to withdraw at any time without penalty. Inclusion criteria were: 1) currently a registered primary school student and one of his/her guardians, 2) aged between 6 and 13, 3) accessible to Facebook through electronic devices at least once a week and 4) able to read and listen to basic Chinese and Cantonese. Excluding criteria were: 1) students suffering from any disease or injury that prevents them from exercising normally, and 2) participating in any activities that may alter the students’ PA routine, e.g., other intervention studies or PA mediators, and 3) guardians refusing to contribute activity time with children at home.

Sample size estimation was conducted using G∗Power software (version 3.1.9.7, Germany). The study was initially powered based on parameters from previous two-arm online exercise interventions,^19^^,^^26^ a minimum of 29 dyads per group was required detect a large between-group effect size (d = 0.75), with 80% power at an alpha level of 0.05, To adequately power the interaction effects within the four-arm RCT design, a 4-fold increase in the total sample was applied according to established recommendation.^38^ According for an anticipated attrition rate of 20%, the final recruitment was set at 145 child-guardian dyads. A total of 140 dyads voluntarily consented. Following initial screening, 119 children proceeded to baseline assessment. Outcome assessors and data analysts were fully blinded to group allocation; however, because this was a behavioral intervention, participants and the research assistant delivering the materials could not be blinded to the group assignments. Participants were randomly allocated into four groups using computer-generated randomization by an independent researcher who was not involved in this study: (i) Exercise Only Group (n = 29), (ii) Coaching Only Group (n = 30), and (iii) Exercise-plus-Coaching Group (n = 31), compared with (iv) a Waitlist Control Group (Control, n = 29). Group allocation was fully concealed from the research team responsible for assessments throughout the intervention period to minimize bias. Ethical approval was obtained from the Human Research Ethics Committee of the authors' institution (Ref. no. 2019-2020-0486). All procedures adhered to the principles outlined in the Declaration of Helsinki, ensuring the protection of participants’ rights, safety, and well-being.

### 2.2 Experimental design

**Fig. 1:**
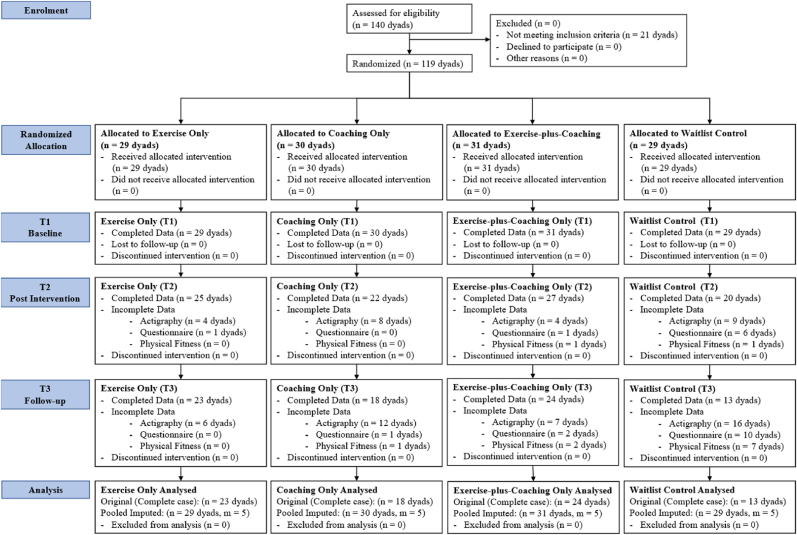
CONSORT Flow Chart of the Study. *Note:* An intention-to-treat approach was followed. Participants with complete baseline data were included in the analysis, and missing outcome data at T2 and T3 were addressed using multiple imputations via Fully Conditional Specification (FCS).

This four-arm single-blinded randomized controlled trial (RCT) involved three online intervention groups and one control (Fig. 1),^39^ and was conducted in accordance with the Consolidated Standards of Reporting Trials (CONSORT) guidelines.^40^ To maintain focus on the primary research questions, this manuscript reports on behavioral (MVPA) and motivational outcomes; results regarding physical fitness and guardian relationship variables are reserved for a separate secondary analysis.

Participants were assessed at three time points within the same academic year: baseline (T1), immediately after 8 weeks of intervention (T2), and 8 weeks post-intervention (T3), to assess the sustainability of effects. To minimize contamination, intervention materials were delivered through private, group-specific Facebook pages. All groups received content with matched frequency and format to reduce expectancy effects. Participants were instructed not to share content or discuss their involvement with others. Given the low-risk nature of the intervention, no interim analyses or formal stopping guidelines were established.

Participants completed three 1-day assessments at their schools at T1, T2, and T3. During these sessions, psychological and motivational surveys were administered via standardized paper-and-pencil questionnaires; trained researchers were present to read questions aloud to younger children to ensure comprehension and accuracy. Additionally, each session included standardized measurements of anthropometric data, body composition, and physical fitness. All assessments were administered in person by trained research staff in a quiet school setting.

### 2.3 Intervention protocol

The intervention was structured according to the Template for Intervention Description and Replication (TIDieR) checklist. Content was delivered twice weekly via private Facebook pages administered by a trained research assistant. On each delivery date, two separate posts were published, totaling four posts per week. Over the eight-week intervention period, each group-specific Facebook page published 32 scheduled posts (comprising one video per post). Intervention engagement was quantified using Facebook post view counts (average views per post), while intervention fidelity was defined a priori as the successful delivery of all scheduled posts.

Guardians accessed group-specific pages: the (i) Exercise Only Group received only home-exercise videos; the (ii) Coaching Only Group received only SDT-informed coaching-strategy videos; the (iii) Exercise-plus-Coaching Group received both; the (iv) Waitlist Control Group received no content during the trial.

Before the intervention, guardians attended a brief face-to-face orientation to familiarize themselves with the Facebook interface and gain confidence in using the materials. The home-exercise videos (1–2 min each) were designed by sport-science professionals to promote home-based MVPA and were tailored for age-appropriateness, safety, and feasibility using common household equipment. The SDT-based coaching materials were delivered as short instructional videos (1–2 min each) accompanied by text-and-image posts. These cues guided guardians to support autonomy (e.g., offering choices), competence (e.g., providing specific, skill-focused feedback), and relatedness (e.g., acknowledging children's effort). For the Exercise-plus-Coaching group, these two components were integrated: each online post was preceded by a specific coaching video delivered immediately prior to the exercise video.

To ensure group isolation, interaction within the Facebook platform was restricted to ‘likes’ or comments directed at the research assistant, while peer-to-peer discussion across groups was discouraged. Although guardians were encouraged to share photos or videos of home activities as part of their individual reporting, no guardian-generated posts were received during the intervention period. All intervention sessions were designed to be completed asynchronously at home, with guardians facilitating the activities. The Control group received no intervention during the study period. To minimize expectancy bias, these participants were blinded to their status until the completion of T3, at which point the intervention videos were shared with them via their schools. Materials were adapted from a validated protocol^41^ shown to enhance motivation and PA engagement in children.^42^ Further details and examples of the exercise and coaching cue content are presented in Table 1 and Table 2.

**Table 1:** Strategies, i.e. SDT-informed online intervention, provided to the experimental group(s).

Intervention component	Target and Expected SDT-based outcomes	Strategies Characteristics & Designed Criteria	Example Strategies & Delivery Format
Parental coaching cues	Autonomy/Competence/Relatedness	**Focus:** To promote autonomous supportive approach in parent-led physical activities. To build guardians' fundamental coaching technique and understanding of exercise motivation. To enhance the guardian's perceived ability to coach and help children exercise. **Selection Criteria:** Grounded in empirical research on teaching behaviors and relational support.	**Format:** Delivered via live-action and animated videos accompanied by text/image posts. **Examples:** • Providing supportive instruction. • Challenge children within their capabilities. • Allowing flexible choice and effort. • Acknowledge children's efforts and difficulties. • Adopting non-judgmental language.
Online exercise options	Autonomy/Competence/Relatedness	**Focus:** To accommodate children with various fitness and commitment levels. To supplement exercise options with written instructions and videos. **Selection Criteria (5 Key Pillars):** Activities selected based on motives, play format, grouping types, movement nature, and spatial feasibility to ensure household adaptability.	**Format:** 1–2 min video demonstrations focusing on the mechanics of how to play the games and the specific steps of each physical activity. **Examples:** • Activities for 1–3 participants, matching local family sizes and the study's dyad structure. • Low-equipment tasks (e.g., balloon tennis, indoor obstacles) tailored for limited urban living spaces.
Facebook project's page	Relatedness	To develop social support and rapport among the guardians.	**Examples:** Encouraging guardians to share photos/videos, like/comment on posts, and providing individualized feedback.

**Table 2:** Overview of intervention components and digital delivery Dosage across experimental and control groups.

Intervention component	Groups				
	Coaching Only	Exercise Only	Exercise-plus-Coaching		Waitlist Control
Parental Coaching cues	√ (32 posts, including: • 10 autonomy focused videos, • 13 competence focused videos, and • 9 relatedness focused videos)	-	✓	(32 posts, Combination)	-
Online Learning Material	-	√ (32 posts)	✓		-
Facebook project's page	✓	✓	✓		-

### 2.4 Measurements

To ensure data integrity, outcome assessments were conducted by two trained research assistants who were blinded to group allocation. Potential harms were monitored informally through direct feedback from guardians and teachers; no serious adverse events were reported during the study period.

#### 2.4.1 Primary outcomes

*Physical activity level.* Participants’ PA levels and sleep behaviours were assessed using the ActiGraph wGT3X-BT triaxial monitor (Pensacola, Florida, USA), worn on the non-dominant wrist for 7 consecutive days. Valid data required a minimum of 10 h of wear-time per day for at least 4 days (including one weekend day). The device has demonstrated reliability for weekly PA tracking (ICC = 0.49–0.67)^43^ and accuracy in detecting awake (95.7%) and sleep time (90.2%) in young children.^44^ Guardians were trained on device use and instructed to log any non-wear periods (e.g., swimming or bathing) in a paper diary.^45^

Data were collected in 10-s epochs, downloaded via ActiLife software, and processed using KineSoft.^46^ Activity cut-points followed Chandler et al.^47^ Outcomes included time spent in MVPA, light physical activity (LPA), sedentary behaviour (SEB), and total step counts across weekdays and weekends. Sleep data were collected for descriptive purposes but excluded from analysis due to space constraints.

*Motivation and Psychological Need Satisfaction.* Children's motivation and psychological need satisfaction were developed and assessed using scales adapted from Sebire et al. (2013), which were specifically for use in primary school-aged children.^46^ Chinese versions were translated from English using a rigorous forward–backward translation procedure.^46^ Two independent native Chinese speakers: one familiar with the constructs and one unfamiliar conducted the forward translation, followed by backward translation by two bilingual translators unfamiliar with the original scales. The final items were pilot-tested with children and reviewed by primary school teachers to ensure clarity and age-appropriateness.^20^^,^^48^

The questionnaire comprised three sections. I) Behavioural Regulation in Exercise Questionnaire. This 12-item scale comprises three questions for each of the four motivation subscales. In the current sample, internal consistency (Cronbach's α) was as follows: intrinsic (α = 0.65), identified (α = 0.66), introjected (α = 0.50) and external motivation (α = 0.54). Amotivation and integrated regulation were excluded, as research indicates that children in this developmental stage do not typically exhibit these characteristics.^46^ Students rated each item on a five-point Likert scale, ranging from 1 “not true for me” to 5 “very true for me.” II) Basic Psychological Needs in Sports and Physical Activity Scale. This 16-item scale assesses autonomy (5 items; α = 0.69), competence (5 items; α = 0.63) and relatedness (6 items; α = 0.68). Students rated each item on a five-point Likert scale, where 1 is “not like me at all” and 5 is “really like me.” III) Perceived Autonomy Support. A 6-item version of Learning Climate Questionnaire was used (α = 0.87). Responses were given using a 7-point Likert scale, ranging from 1 “strongly disagree” to 7 “strongly agree”. Average subscale scores were used to assess motivational profiles across groups.

#### 2.4.2 Secondary outcomes

To maintain a focused analysis on the primary outcomes of child MVPA and motivation, several secondary measures collected during the trial are not reported in the current paper. These include anthropometric data (height, weight, and body composition), muscle strength (handgrip strength using a digital dynamometer), and guardian-related measures (self-reported PA habits and perceived parent-child relationship quality).

Cardiorespiratory Fitness was assessed using a modified Yo-Yo IR1 test,^49^ with children shuttling between 16-m lines for 5 min, guided by standardized audio cues. To ensure accuracy and motivation, children performed the test in groups of eight. A trained research assistant ran with each group to help the children maintain the correct pace and provide motivation. Children wore numbered bibs; when a participant was unable to complete a shuttle for the second time, their bib number was announced, and the final completed level and distance were recorded by a second observer. All fitness and guardian-related data are reserved for separate analysis and are not reported in the current results.

### 2.5 Statistical analysis

All statistical analyses were conducted using IBM SPSS Statistics version 28.0 (IBM, Armonk, NY) following intention-to-treat principles. A two-tailed significance level of 5% was applied throughout. Descriptive statistics were calculated for demographic and outcome variables. Baseline comparisons used univariate ANOVAs for continuous variables and chi-square tests (χ^2^) for categorical variables. To evaluate intervention effects, a two-way mixed ANOVA was performed with time (within-subject) and group (between-subject) as factors. Effect sizes were reported as partial eta-squared (η^2^) for ANOVAs and Cohen's d for t-tests. Thresholds for interpretation were: η^2^ = 0.01 (small), 0.06 (medium), 0.14 (large)^50^; d = 0.2 (small), 0.6 (medium), 1.2 (large).^51^ Bonferroni corrections were applied where necessary to control for Type I error.

Missing data were addressed using multiple imputations via the Fully Conditional Specification (FCS) method. The imputation model included baseline predictors (e.g., MVPA, LPA, SEB at T1), with T2 and T3 outcomes serving as both predictors and targets. Imputed values were constrained (0–1440 min/day), and five datasets were generated using 50 case draws and 2 parameter draws per iteration to ensure convergence. Pooled estimates were used in subsequent inferential analyses. This imputation strategy was also applied to psychological scale data.

## 3 Results

### 3.1 Participants

Baseline characteristics of the 119 child-guardian dyads (child mean age = 7.36 ± 0.95 years) enrolled in the 8-week intervention are presented in Table 3a, Table 3b. The participating guardians were primarily mothers (77.3%), with 39.5% in full-time employment and 35.3% currently unemployed. At baseline (T1), children engaged in 34.61 ± 19.48 min of MVPA per day, which falls below the WHO's recommendation of 60 min daily for this age group, indicating insufficient habitual PA.

**Table 3a:** Children's participant characteristics measured at T1 (n = 119).

	Waitlist Control (n = 29)	Exercise Only (n = 29)	Coaching Only (n = 30)	Exercise-plus-Coaching (n = 31)	Total (n = 119)
**Demographics**					
Age, yr	7.52 ± 0.87	7.31 ± 0.97	7.30 ± 1.06	7.32 ± 0.95	7.36 ± 0.95
Sex (male/female), n	20/9	13/16	17/13	17/14	67/52
Height, cm	128.31 ± 6.74	125.28 ± 7.39	123.73 ± 8.28	127.53 ± 7.86	126.71 ± 7.60
Weight, kg	27.75 ± 7.71	25.03 ± 5.65	26.27 ± 8.15	25.68 ± 5.49	26.18 ± 6.83
Body mass index, kg/m^2^	16.61 ± 3.00	15.77 ± 2.25	16.31 ± 3.10	15.63 ± 1.99	16.08 ± 2.62
Percentage of body fat (%)	16.23 ± 8.86	14.50 ± 6.54	15.58 ± 8.78	13.61 ± 6.27	14.96 ± 7.66
**Physical Fitness**					
Handgrip strength, kg	22.90 ± 5.61	19.78 ± 6.25	21.95 ± 6.57	21.06 ± 4.42	21.42 ± 5.80
Handgrip strength (Right), kg	11.80 ± 3.11	10.12 ± 3.21	11.13 ± 3.59	10.79 ± 2.26	10.96 ± 3.09
Handgrip strength (Left), kg	11.10 ± 2.78	9.66 ± 3.17	10.82 ± 3.24	10.27 ± 2.36	10.46 ± 2.92
Yoyo test level 1 – Children (m)	230.62 ± 220.28	218.48 ± 133.06	254.93 ± 155.29	245.68 ± 176.90	237.71 ± 172.61
Maximum heart rate during Yoyo test, bpm	177.93 ± 27.81	166.28 ± 24.69	168.03 ± 25.51	163.16 ± 26.18	168.75 ± 26.32
**Physical Activity Level (4+ valid days)**					
Time spent MVPA (min)	43.19 ± 22.43	28.34 ± 16.79∗	36.68 ± 18.74	30.45 ± 17.03	34.61 ± 19.48
Weekday (min)	31.77 ± 18.45	27.25 ± 17.01∗	35.83 ± 19.18	29.21 ± 16.98	33.28 ± 19.32
Weekend (min)	48.57 ± 35.34	31.103 ± 28.29	38.78 ± 27.65	33.57 ± 25.68	37.94 ± 29.77
Time spent LPA (min)	271.68 ± 74.87	254.16 ± 86.34	271.58 ± 76.24	254.24 ± 68.98	262.84 ± 76.25
Weekday (min)	280.66 ± 85.12	255.60 ± 97.67	280.77 ± 80.40	258.53 ± 77.74	268.82 ± 85.14
Weekend (min)	249.24 ± 73.69	250.53 ± 88.09	248.60 ± 99.82	243.50 ± 78.44	247.90 ± 84.56
Time spent sedentary (SED, min)	1125.05 ± 88.95	1154.12 ± 97.76	1131.75 ± 86.23	1150.72 ± 87.38	1140.51 ± 89.81
Weekday (min)	1118.19 ± 100.45	1152.42 ± 107.62	1123.40 ± 91.88	1145.83 ± 102.03	1135.05 ± 100.37
Weekend (min)	1142.19 ± 86.73	1158.36 ± 105.81	1152.62 ± 109.89	1162.94 ± 90.31	1154.16 ± 97.69
Average daily step count (step)	12755.72 ± 3113.20	11098.11 ± 3696.60	12149.50 ± 2788.81	11657.60 ± 2631.69	11912.87 ± 3095.74
Weekday (step)	12815.42 ± 3408.47	11101.58 ± 4065.55	12431.55 ± 2985.08	11665.26 ± 3052.69	12001.37 ± 3417.09
Weekend (step)	12606.47 ± 3481.77	11089.45 ± 4024.31	11444.35 ± 3959.32	11638.45 ± 3316.70	11691.63 ± 3698.64
**Sleep**					
Time in bed (min)	448.89 ± 92.60	484.01 ± 140.55	485.51 ± 165.60	487.38 ± 150.52	476.71 ± 139.55
Weekday (min)	434.68 ± 88.60	473.72 ± 154.77	476.18 ± 192.69	481.82 ± 208.60	466.94 ± 167.58
Weekend (min)	435.85 ± 98.34	520.60 ± 215.54	520.90 ± 210.54	523.52 ± 196.97	496.38 ± 192.92
Total sleeping time (min)	410.68 ± 97.74	446.94 ± 140.02	454.40 ± 172.08	446.02 ± 149.51	439.74 ± 142.01
Weekday (min)	395.20 ± 91.67	437.76 ± 156.33	439.34 ± 199.37	418.60 ± 215.00	422.80 ± 172.13
Weekend (min)	402.90 ± 99.77	483.60 ± 220.42	484.00 ± 216.58	481.70 ± 196.27	459.50 ± 194.64

Means ± standard deviations are presented unless specified otherwise. Analyze all data and report the valid data only.

∗*p* < .05 compared with Control.

**Table 3b:** Children's participant characteristics measured at T1 (n = 119).

	Waitlist Control (n = 29)	Exercise Only (n = 29)	Coaching Only (n = 30)	Exercise-plus-Coaching (n = 31)	Total (n = 119)
**Behavioural Regulation in Exercise Questionnaire**^a^					
Intrinsic Motivation	4.38 ± 0.61	4.22 ± 0.87	4.17 ± 0.95	4.15 ± 0.67	4.23 ± 0.78
Identified Regulations	4.00 ± 0.90	3.90 ± 0.98	4.12 ± 1.01	4.27 ± 0.81	4.08 ± 0.93
Introjected Regulations	3.26 ± 1.13	3.36 ± 1.02	3.40 ± 1.17	3.51 ± 1.09	3.38 ± 1.09
External Motivation	3.00 ± 1.08	2.74 ± 1.03	2.87 ± 1.44	2.69 ± 0.98	2.82 ± 1.14
**Basic Psychological Needs in Sports and Physical Activity Scale**^b^					
Autonomy	3.93 ± 0.82	4.08 ± 0.87	3.91 ± 0.82	4.05 ± 0.92	3.99 ± 0.85
Competence	3.79 ± 0.81	4.12 ± 0.76	4.05 ± 0.68	3.99 ± 0.76	3.98 ± 0.76
Relatedness	3.80 ± 0.75	4.03 ± 0.79	3.78 ± 0.79	3.88 ± 0.76	3.87 ± 0.77
**Learning Climate Questionnaire**^c^					
Perceived Autonomy Support from Parents	5.27 ± 1.34	5.40 ± 1.26	5.63 ± 1.33	5.61 ± 1.67	5.48 ± 1.41

Means ± standard deviations are presented unless specified otherwise. Analyze all data and report the valid data only.

a 5-Point Likert Scale (1 “not true for me” to 5 “very true for me.”).

b 5-Point Likert Scale (1 is “not like me at all” and 5 is “really like me.”).

c 7-Point Likert Scale (1 “strongly disagree” to 7 “strongly agree”); ∗*p* < .05.

Motivational profiles assessed at T1 revealed consistently high levels of intrinsic motivation across all groups, with a mean score of 4.23 ± 0.78 on a five-point Likert scale, suggesting a strong internal desire to engage in PA. Children also reported satisfactory levels of basic psychological need satisfaction, with autonomy, competence, and relatedness scores ranging from 3.87 to 3.99 out of 5, reflecting a generally positive motivational climate.

Perceived autonomy support from guardians was also favorable, with a mean score of 5.48 ± 1.41 on a seven-point Likert scale, indicating that children felt encouraged and supported in their PA choices. In terms of physical health status, participants’ body mass index values placed them within the normal weight range, and no significant between-group differences were observed at baseline (T1).

### 3.2 Actigraphy data across time

Actigraphy data were considered valid only if at least four days of actigraphy data were collected. The analysis included both original and imputed datasets to address missing data, which ranged from 21.01% (n = 22) at T2 to 34.45% (n = 41) at T3, with an original sample size of 119 participants at T1 (Table 4a).

**Table 4a:** Pooled Imputed Actigraphy Data (n = 119, m = 5).

Groups		T1			T2			T3			Time Effect	
											T2 – T1	T3 – T1
**MVPA (min) ∗^, t^**												
Control	(n = 29, m = 5)^t^	43.19	±	22.11	35.37	±	18.59	29.02	±	16.89	^d = −0.44	^d = −0.66
Exercise Only	(n = 29, m = 5)^t^	28.35	±	16.56	34.23	±	20.95	29.80	±	19.48	^d = 0.36	NS
Coaching Only	(n = 30, m = 5)^t^	36.68	±	18.48	38.14	±	23.07	33.55	±	16.86	NS	NS
Exercise-plus-Coaching	(n = 31, m = 5)^t^	30.45	±	16.81	37.37	±	16.67	33.02	±	16.42	^d = 0.39	NS

LPA (min) ∗^,t^												
Control	(n = 29, m = 5)^t^	271.68	±	73.83	275.14	±	70.87	247.92	±	82.06	NS	^d = −0.31
Exercise Only	(n = 29, m = 5)	254.16	±	85.13	257.27	±	70.93	255.56	±	68.31	NS	NS
Coaching Only	(n = 30, m = 5)^t^	271.58	±	75.21	267.19	±	83.06	246.42	±	77.20	NS	^d = −0.32
Exercise-plus-Coaching	(n = 31, m = 5)^t^	254.24	±	68.08	269.33	±	65.90	248.90	±	73.34	^d = 0.26	NS

SEB (min) ∗												
Control	(n = 29, m = 5)^t^	1125.05	±	87.71	1129.05	±	87.71	1075.15	±	172.65	NS	^d = −0.31
Exercise Only	(n = 29, m = 5)	1154.12	±	96.40	1149.95	±	82.46	1155.30	±	104.47	NS	NS
Coaching Only	(n = 30, m = 5)^t^	1131.75	±	85.07	1134.96	±	97.51	1158.17	±	103.41	NS	^d = 0.25
Exercise-plus-Coaching	(n = 31, m = 5)^t^	1150.72	±	86.24	1137.47	±	78.93	1163.93	±	100.78	NS	NS

Mean ± SD; ∗ Group × Time Intervention effect (*p* < .05); ^t^ Time Effect; ^ Time effect compared to T1 with Bonferroni adjustment (*p* < .05); NS: No significant difference.

The interaction effect between time and group was statistically significant for MVPA and SEB across all datasets. For the pooled imputed dataset, the multivariate tests indicated significant interactions for MVPA (*p* < .001, partial η^2^ = 0.069), and for SEB, (*p* < .001, partial η^2^ = 0.053). Although LPA demonstrated a significant interaction effect (*p* = .003) in the pooled imputed data, it only has a partial η^2^ of 0.016, indicating a negligible effect size.

In the pooled imputed dataset, a significant Group × Time interaction effect was observed. Pairwise comparison with Bonferroni adjustment revealed that the minutes of MVPA increased from T1 to T2 in the (i) Exercise Only Group and the (iii) Exercise-plus-Coaching Group by 5.88 min (d = 0.36) and 6.91 min (d = 0.39), respectively, indicating small-to-medium effects at T2; both returned to approximately baseline levels at T3. Similar results were observed in the original datasets, confirming the consistency of this finding. Conversely, MVPA in the Control group decreased significantly from T1 to T2, with a reduction of 7.82 min (*p* < .001), and further decreased by 14.17 min at T3 (*p* < .001).

### 3.3 Children's physical activity motivation and psychological need satisfaction

For all questionnaire scales, both original and imputed datasets were used to address missing data, which ranged from 6.72% (n = 8) at T2 to 10.92% (n = 13) at T3, based on an initial sample of 119 participants at T1 (see Table 4b).

**Table 4b:** Pooled Imputed Children: a) Physical activity motivation scales and b) Psychological need satisfaction scales across time (n = 119, m = 5).

		T1			T2			T3			Time Effect	
											T2 – T1	T3 – T1
**Behavioural Regulations in Exercise Questionnaire**^a^												
Intrinsic Motivation **∗^, ^^**												
Control	(n = 29, m = 5) **^t^**	4.38	±	0.60	4.16	±	0.79	3.68	±	0.77	^d = −0.28	^d = −0.88
Exercise Only	(n = 29, m = 5)	4.22	±	0.85	4.23	±	0.79	4.31	±	0.79	NS	NS
Coaching Only	(n = 30, m = 5) **^t^**	4.17	±	0.94	4.30	±	0.65	4.49	±	0.63	NS	^d = 0.29
Exercise-plus-Coaching	(n = 31, m = 5)	4.15	±	0.66	4.15	±	0.83	4.03	±	0.77	NS	NS
Identified Regulation **∗^, ^^**												
Control	(n = 29, m = 5) **^t^**	4.00	±	0.89	3.78	±	0.89	3.43	±	0.82	^d = −0.20	^d = −0.61
Exercise Only	(n = 29, m = 5)	3.90	±	0.97	3.95	±	0.82	3.89	±	1.00	NS	NS
Coaching Only	(n = 30, m = 5) **^t^**	4.12	±	0.99	4.11	±	0.72	4.30	±	0.73	NS	NS
Exercise-plus-Coaching	(n = 31, m = 5) **^t^**	4.27	±	0.80	4.24	±	0.87	3.83	±	0.96	NS	^d = −0.47
Introjected Regulation **∗^, ^^**												
Control	(n = 29, m = 5) **^t^**	3.26	±	1.12	2.42	±	0.88	2.86	±	0.95	^d = −0.68	^d = −0.28
Exercise Only	(n = 29, m = 5) **^t^**	3.36	±	1.01	2.76	±	1.06	3.25	±	1.00	^d = −0.45	NS
Coaching Only	(n = 30, m = 5) **^t^**	3.40	±	1.15	2.52	±	1.07	3.44	±	0.98	^d = −0.55	NS
Exercise-plus-Coaching	(n = 31, m = 5) **^t^**	3.51	±	1.07	2.78	±	1.24	3.19	±	0.91	^d = −0.56	^d = −0.31
External Regulation **∗^, ^^**												
Control	(n = 29, m = 5) **^t^**	3.00	±	1.07	2.42	±	0.88	2.22	±	0.76	^d = −0.49	^d = −0.66
Exercise Only	(n = 29, m = 5)	2.74	±	1.01	2.76	±	1.06	2.69	±	0.84	NS	NS
Coaching Only	(n = 30, m = 5) **^t^**	2.87	±	1.42	2.52	±	1.07	2.70	±	1.03	^d = −0.22	NS
Exercise-plus-Coaching	(n = 31, m = 5) **^t^**	2.69	±	0.97	2.78	±	1.24	2.55	±	1.00	NS	NS

**Basic Psychological Needs in Sports and Physical Activity Scale**^b^												

Autonomy **∗^, ^^**												
Control	(n = 29, m = 5) **^t^**	3.93	±	0.81	3.86	±	0.79	3.75	±	0.69	NS	^d = −0.23
Exercise Only	(n = 29, m = 5)	4.08	±	0.86	4.16	±	0.70	4.16	±	0.77	NS	NS
Coaching Only	(n = 30, m = 5) **^t^**	3.91	±	0.81	4.20	±	0.69	4.31	±	0.67	^d = 0.30	^d = 0.47
Exercise-plus-Coaching	(n = 31, m = 5) **^t^**	4.05	±	0.91	4.09	±	0.61	3.85	±	0.72	NS	^d = −0.22
Competence **∗^, ^^**												
Control	(n = 29, m = 5) **^t^**	3.79	±	0.80	3.52	±	0.87	3.70	±	0.80	^d = −0.25	NS
Exercise Only	(n = 29, m = 5) **^t^**	4.12	±	0.74	3.70	±	0.85	3.89	±	0.71	^d = −0.41	^d = −0.25
Coaching Only	(n = 30, m = 5)	4.05	±	0.67	4.11	±	0.58	3.99	±	0.81	NS	NS
Exercise-plus-Coaching	(n = 31, m = 5) **^t^**	3.99	±	0.75	3.96	±	0.85	3.69	±	0.95	NS	^d = −0.36
Relatedness **∗^, ^^**												
Control	(n = 29, m = 5)	3.80	±	0.74	3.79	±	0.77	3.73	±	0.72	NS	NS
Exercise Only	(n = 29, m = 5)	4.03	±	0.78	3.95	±	0.77	4.04	±	0.87	NS	NS
Coaching Only	(n = 30, m = 5) **^t^**	3.78	±	0.78	4.14	±	0.63	4.23	±	0.64	^d = 0.42	^d = 0.52
Exercise-plus-Coaching	(n = 31, m = 5) **^t^**	3.88	±	0.75	4.17	±	0.72	4.06	±	0.74	^d = 0.33	^d = 0.21

**Learning Climate Questionnaire**^c^												
Perceived Autonomy Support **^t^**												
Control	(n = 29, m = 5)	5.27	±	1.32	5.43	±	1.18	5.38	±	1.07	NS	NS
Exercise Only	(n = 29, m = 5)	5.40	±	1.24	5.31	±	1.54	5.48	±	1.42	NS	NS
Coaching Only	(n = 30, m = 5)	5.63	±	1.32	5.77	±	1.18	5.83	±	1.10	NS	NS
Exercise-plus-Coaching	(n = 31, m = 5) **^t^**	5.61	±	1.65	5.95	±	0.86	5.83	±	1.12	**^^^**d = 0.21	NS

Mean ± SD; ∗ Group × Time Intervention effect (*p* < .05); ^t^ Time Effect; ^ Time effect compared to T1 with Bonferroni adjustment (*p* < .05).

NS: No significant difference. “m” denotes the number of multiply imputed datasets (m = 5) used to address missing data.

a 5-Point Likert Scale (1 “not true for me” to 5 “very true for me.”).

b 5-Point Likert Scale (1 is “not like me at all” and 5 is “really like me.”).

c 7-Point Likert Scale (1 “strongly disagree” to 7 “strongly agree”).

In the original dataset, significant interaction effects between time and group were found for intrinsic motivation (p = .017, partial η^2^ = 0.075) and autonomy needs (p = .045, partial η^2^ = 0.063). In the pooled imputed datasets, interaction effects were significant for the motivation scales and psychological need satisfaction, but not for perceived autonomy support from parents, indicating differential trajectories by group.

A significant main effect of time was observed across all scales in the imputed data. Effect sizes ranged from small (partial η^2^ = 0.006 for autonomy) to large (partial η^2^ = 0.152 for introjected regulation). Children in the intervention groups maintained high levels of intrinsic motivation over time, while the Control group showed a steady decline in both intrinsic motivation and identified regulation, with small effects at T2 (d = −0.28 and −0.20) and large effects at T3 (d = −0.88 and −0.61).

For introjected regulation, all groups declined at T2 compared to T1, but by T3, scores either returned to baseline or showed less reduction. External regulation remained stable in the intervention groups, except for Coaching Only Group, which showed a small decrease. The Control group showed a moderate decline in external regulation at both T2 and T3.

Autonomy was higher in Coaching Only Group at T2 (d = 0.30) and continued to improve at T3 (d = 0.47), while other groups declined at T3. Competence decreased at T2 in the Exercise Only and Control groups but remained stable in Exercise-plus-Coaching and Coaching Only groups. Relatedness increased in both groups receiving coaching cues at T2 and T3, a change not observed in Exercise Only Group or Control. Overall, children reported consistently high levels of perceived autonomy support from parents, with minimal differences between groups or across time.

### 3.4 Implementation (engagement and fidelity)

Across the 32 scheduled posts, average Facebook views per post were higher for exercise content than for coaching content: Exercise Only Group 9.41 ± 3.71 views per post; Exercise-plus-Coaching Group (coaching page) 8.94 ± 3.18 views per post; Coaching Only Group 6.47 ± 2.18 views per post. All 32 scheduled posts were delivered on each intervention page, meeting the predefined fidelity standard.

## 4 Discussion

This study provides preliminary evidence for the short-term effectiveness of an SDT-informed, guardian-led online intervention in promoting PA and motivation among primary school-aged children. Children in the Exercise Only and Exercise-plus-Coaching groups demonstrated significantly higher MVPA levels than the Control group post-intervention, highlighting the potential of scalable, low-cost, theory-driven online programs to support home-based PA. However, as these differences were not sustained at the 8-week follow-up, the evidence suggests an acute rather than a long-term impact on behavior.

Importantly, the intervention successfully preserved children's intrinsic motivation and identified regulation, counteracting the decline observed in the Control group. This finding aligns with SDT's assertion that autonomous motivation is critical for sustained engagement in PA.^52^ Notably, children whose guardians received coaching cues, either alone or alongside activity choices, reported greater satisfaction in autonomy, competence, and relatedness, reinforcing the value of autonomy-supportive parenting.

In terms of time effects, the MVPA decline in the Control group mirrors previous research. For instance, Dishman et al.^53^ reported a 4.03 min annual drop in daily MVPA among sixth graders. In contrast, our study found a sharper decline of 14.17 min over eight weeks among younger children in Hong Kong, underscoring the urgency of early intervention. While prior studies have focused on teacher-led PE, e.g. Ha et al.^42^ and Teixeira et al.,^52^ our findings extend this evidence by demonstrating that guardian involvement can effectively buffer against the decline in PA levels observed in non-intervention settings.

Furthermore, the role of adult-child interactions appears central to short-term behavioral maintenance. Van Doren et al.^54^ cautioned that controlling teaching styles may increase short-term in-class MVPA but undermine intrinsic motivation. In the context of Hong Kong, parenting is often characterized by an authoritative or demanding style driven by high academic expectations. This intervention provided guardians with a framework for autonomy-supportive interaction, which may serve as a vital alternative to traditional demanding styles that often discourage voluntary PA. Our findings suggest that such interactions, particularly those facilitated by guardians, are more conducive to maintaining activity levels during the intervention period.

Despite the positive outcomes, MVPA levels declined once the intervention ended, even though autonomous motivation was retained. This suggests that while motivation was internalized, it was insufficient to overcome environmental barriers without the ongoing structure provided by the digital platform. Therefore, future interventions should incorporate continuous support mechanisms, such as school-linked online platforms and periodic booster sessions. The Facebook platform served not only as a delivery tool but as a resource for social support among guardians; future research should specifically investigate how digital communities of parents can reinforce peer-based encouragement to sustain long-term engagement.

The intervention's design, which combined structured activity choices with SDT-informed coaching strategies, proved particularly effective. Activities were selected based on five key criteria: activity motives, play format, grouping types, movement nature, and spatial feasibility, to ensure adaptability across diverse household environments. Guardians received two posts per delivery day, twice weekly, offering flexible options to encourage participation. However, when coaching cues were absent, children reported lower psychological need satisfaction, and in some cases, diminished competence. This may reflect inconsistent or controlling coaching styles, which resemble the demanding or disengaged teaching approaches often observed in school PE settings.^54^

Importantly, our findings align with recent studies,^55^, ^56^, ^57^ which emphasize that integrating SDT-informed strategies with practical tools enhances intervention effectiveness. The coaching cues, delivered in both live-action and animated formats, were grounded in empirical research on teaching behaviors, relational support,^58^ and the impact of rewards on motivation,^59^ and effectively addressed children's psychological needs.

Additionally, this study contributes to understanding how parental engagement influences children's PA. Parents who model active behaviors and provide supportive environments significantly enhance children's intrinsic motivation and activity levels.^60^^,^^61^ By targeting guardians rather than children or teachers directly, the intervention created a family-centered motivational climate that fulfilled SDT's core psychological needs and promoted sustainable behavior change.

Nonetheless, several limitations should be acknowledged. The short follow-up period and the focus on acute effects limit our ability to conclude whether these strategies can lead to permanent lifestyle modifications. Additionally, while we collected data on cardiorespiratory fitness, muscle strength, and guardian-child relationship quality, these were reserved for a separate analysis to maintain the focus of this paper. We are currently analyzing these secondary outcomes to provide a more comprehensive report on the intervention's impact on holistic health and family dynamics.

In conclusion, this study demonstrates that brief, SDT-informed, guardian-focused interventions can enhance children's PA and motivation in the short term. While we did not observe permanent behavior change post-intervention, the program effectively mitigated the significant decline in MVPA seen in the Control group. Future efforts should prioritize family-centered strategies that integrate theory with practical delivery to promote behavioral preservation and help reverse the global decline in youth PA.

## Ethics approval statement

The ethical approval for this research was sought from the Human Research Ethics Committee at the Education University of Hong Kong (Ref. no. 2019-2020-0486).

## Author statement

Chi-Ching Gary CHOW secured the funding for the project, led design of the study, data analysis, and writing of the manuscript, with critical input provided by Fenghua SUN, and Yu-Hin KONG. Yu-Hin KONG conducted data analysis. All authors revised the manuscript for intellectual content and approved the final submitted version.

## Declaration of generative AI in scientific writing

During the preparation of this work the author(s) used Generative AI and AI-assisted technologies in order to improve the readability and language of the manuscript. After using this tool/service, the author(s) reviewed and edited the content as needed and take(s) full responsibility for the content of the published article.

## Funding

This study was substantially supported by Early Career Scheme (ECS) 2021/22, Research Grants Council of the Hong Kong Special Administrative Region, China (Grant number: 28602121).

## Declaration of competing interest

Author Fenghua SUN is an Associate Editor of Journal of Exercise Science and Fitness. Fenghua SUN was not involved in the journal's peer review process of, or decisions related to, this manuscript.
